# σ-Aromaticity in planar pentacoordinate aluminium and gallium clusters

**DOI:** 10.1038/s41598-022-14430-4

**Published:** 2022-06-16

**Authors:** Amlan J. Kalita, Kangkan Sarmah, Farnaz Yashmin, Ritam R. Borah, Indrani Baruah, Rinu P. Deka, Ankur K. Guha

**Affiliations:** Department of Chemistry, Advanced Computational Chemistry Centre, Cotton University, Panbazar, Guwahati, Assam 781001 India

**Keywords:** Coordination chemistry, Inorganic chemistry

## Abstract

Planar hypercoordinate structures are gaining immense attention due to the shift from common paradigm. Herein, our high level ab initio calculations predict that planar pentacoordinate aluminium and gallium centres in Cu_5_Al^2+^ and Cu_5_Ga^2+^ clusters are global minima in their singlet ground states. These clusters are thermodynamically and kinetically very stable. Detailed electronic structure analyses reveal the presence of σ-aromaticity which is the driving force for the stability of the planar form.

## Introduction

Introducing new chemical structures is one of the most important goals in chemistry. Challenging the known dogma of chemical structure has always been fascinating in chemical community. One such example of rule breaking structure is the case of planar tetracoordinate carbon (ptC) which was first convincingly proposed in 1968 to exist in planar *D*_4h_ transition states between tetrahedral enantiomers of simple substituted methane^1^. This observation led Hoffmann, Alder, and Wilcox in 1970 to explore the electronic feature for the stabilization of such ptCs by using molecular orbital theory^2^. However, ab initio study revealed that isolation of planar methane is impossible as the energy difference between the planar and tetrahedral form is^3^ 130 kcal/mol. The first global minimum ptCs were predicted in 1976 on some Li-substituted ptC molecules and 1,1-dilithiocyclopropane and 3,3-dilithio-cyclopropene molecules^4^. The first experimental evidence of ptC came in the year^5^ 1977. Since then many planar penta- (ppC), hexa- (phC) and hepta-coordinate (p7C) carbon were computationally explored^6–15^. For example, in 2008, the first ppC, CAl_5_^+^ was experimentally characterized and theoretically found to be global minima^14^. Similarly, the first phC, B_6_C^2^ was proposed by Schleyer et al.^11^ however, the latter study revealed that carbon avoids planar hypercoordination^12^. The true global minimum containing a phC was recently proposed^15^ which contain a planar hexacoordinate carbon atom surrounded by ligands with half covalent and half ionic bonding. Such achievements of novel hypercoordinate carbon (hpC) molecules have created a new dogma in present day chemistry that highest coordination number of carbon in no longer four.

These studies have also inspired the quest for other systems containing planar hypercoordinate main group elements such as group 13 elements. The first molecule containing planar hexacoordinate boron (phB) was predicted in^16^ 1991 which triggered further examples of hypercoordinate boron^16–28^. Zhai et al.^20^ reported experimental and theoretical evidence of perfectly planar B_8_^-^ and B_9_^-^ anions clusters with hepta and octa-coordinate boron atom. Theoretical study by Yu et al.^26^ revealed that B_6_H_5_^+^ cation is aromatic with a planar pentacoordinate boron (ppB) centre. We have also recently reported a phB species, BBe_6_H_6_^+^ cluster featuring double aromaticity^27^. Unlike B, its heavier analogues (Al and Ga) received little attention in the realm of planar hypercoordination. Schleyer and co-workers had shown that there is a dramatic drop in the energy of lowest unoccupied molecular orbital (LUMO) upon planarization of^28^ AlH_4_^−^. Experimental realization of ptAl species has been reported^29,30^. Averkiev et al.^31^ have made an in silico prediction of planar nonacoordinate aluminum centre, AlB_9_, based on previously proved doubly aromatic boron clusters^20^. All boron planar aromatic clusters has also been studied by both theoretically and experimentally^32,33^. A cluster containing a phAl in the global minimum form of Al_4_C_6_ cluster was also reported^34^. Similarly, recent theoretical study has also reported a thermodynamically and kinetically stable phGa species^35^. Despite of these achievements, reports on planar pentacoordinate Al and Ga centres (ppAl and ppGa) in isolated clusters are extremely rare. Herein, we report the global minimum of ppAl and ppGa centres in isolated binary clusters, Cu_5_Al^2+^ and Cu_5_Ga^2+^. The proposed ppAl and ppGa clusters are found to be thermodynamically and kinetically stable and possess double aromaticity.

## Computational details

In order to explore systematically the potential energy surface of the title compound, ABCluster code^36,37^ in combination with M06-2X/TZVP level^38^ has been adopted which produced 30 stationary points. For the exploration of the potential energy surface, both the singlet and triplet states were considered. Full optimization of the low lying isomers was done using M06-2X/def2-TZVP level. The motivation towards using M06-2X functional is due to the fact that this functional has been used to explore potential energy surface of planar pentacoordinate nitrogen (ppN)^39^, planar hexacoordinate boron (ppB)^27^ and ppM (M = Zn, Cd, Hg)^40^. Moreover, to investigate the artifact of the level of theory used, we have further optimized the local minima ppAl and ppGa structures at TPSSh^41^ and PBE0^42^ level. All these provided minimum energy structures with large value of the lowest vibrational frequency (Table S1, supporting information). Hence, the discussion in the text will be based on M06-2X results unless otherwise noted. The energies were then refined by running single point calculations at CCSD(T)/def2-TZVP level of theory on M06-2X optimized geometries. Vibrational harmonic frequency calculations were also performed at M06-2X/def2-TZVP level to confirm that the structures are true minima. All these calculations were performed using Gaussian16 suite of programs^43^. The electronic structure of the molecules were analyzed using natural bond orbital (NBO)^44^ calculations at M06-2X/def2-TZV level. ETS-NOCV analysis were performed at M06-2X/def2-TZVP level considering cationic Al and Ga (triplet) and triplet Cu_5_^+^ as Δ*E*_orb_ for these fragmentation is the least and hence the best choice^45,46^. In addition, adaptive natural density partitioning (AdNDP)^47^, quantum theory of atoms in molecules (QTAIM)^48^ and electron localization function (ELF)^49,50^ were also analyzed at M06-2X/def2-TZV level using Multiwfn program code^51^. To quantify the aromaticity, nucleus independent chemical shift calculations (NICS)^52^ were performed by placing a ghost atom (symbol Bq) at the geometric mean of the Cu-Al-Cu and Cu-Ga-Cu rings and with an incremental distance of 0.2 Å above the ring upto a distance of 2 Å.

## Results and discussion

**Figure 1:**
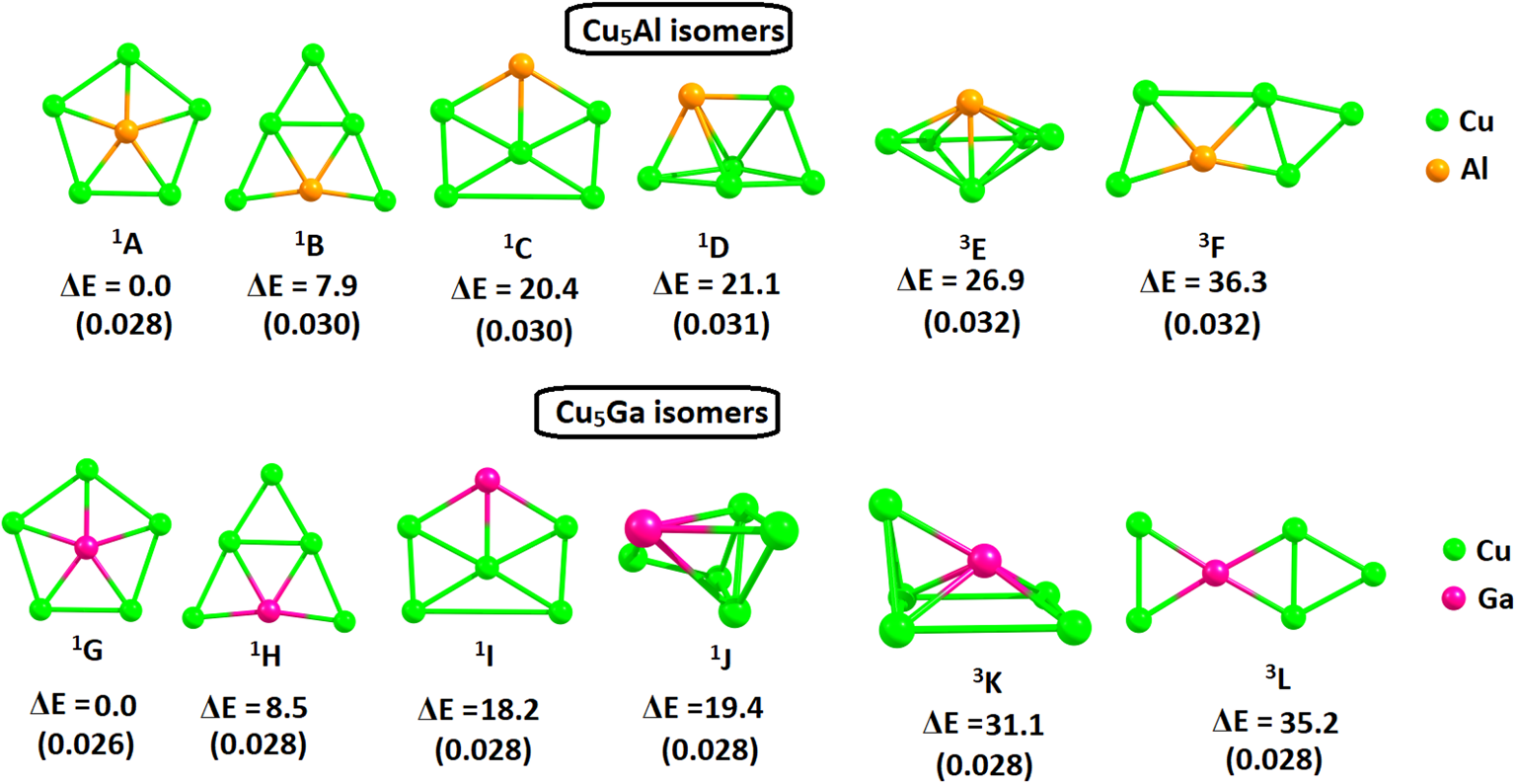
The relative energies in kcal/mol of the low energy isomers (**A**–**L**) of Cu_5_Al^2+^ (top) and Cu_5_Ga^2+^ (bottom) calculated at CCSD(T)/def2-TZVP//M06-2X/def2-TZVP level of theory. T_1_ diagnostic values are given within parenthesis.

Figure 1 shows the optimized geometries of the global minima of Cu_5_Al^2+^ and Cu_5_Ga^2+^ along with their low energy isomers calculated at CCSD(T)/def2-TZVP//M06-2X/def2-TZVP level. The global minima of Cu_5_Al^2+^ (^**1**^**A**) and Cu_5_Ga^2+^ (^**1**^**G**) clusters feature *D*_5h_ symmetry in their singlet spin ground states. The Cu-Al distance in ^**1**^**A** is 2.481 Å (Wiberg bond index is 0.512) while the Cu-Cu distance is 2.917 Å (WIB is 0.052). Similarly, the Cu-Ga distance in ^**1**^**G** is 2.500 Å (WIB is 0.492) and Cu-Cu distances are in the range 2.932–2.942 Å (WIBs are in the range (0.045–0.048). The computed natural charges using NBO method reveals a negative charge of − 1.28e and − 1.37e for the central Al and Ga atoms respectively while Cu atoms are significantly positive. This implies a significant amount of charge donation from the Cu_5_ unit to the central Al and Ga atoms. Moreover, the attraction between these opposite charges may result in high degree of electrostatic interaction which may also account for the stability of these clusters.

**Figure 2:**
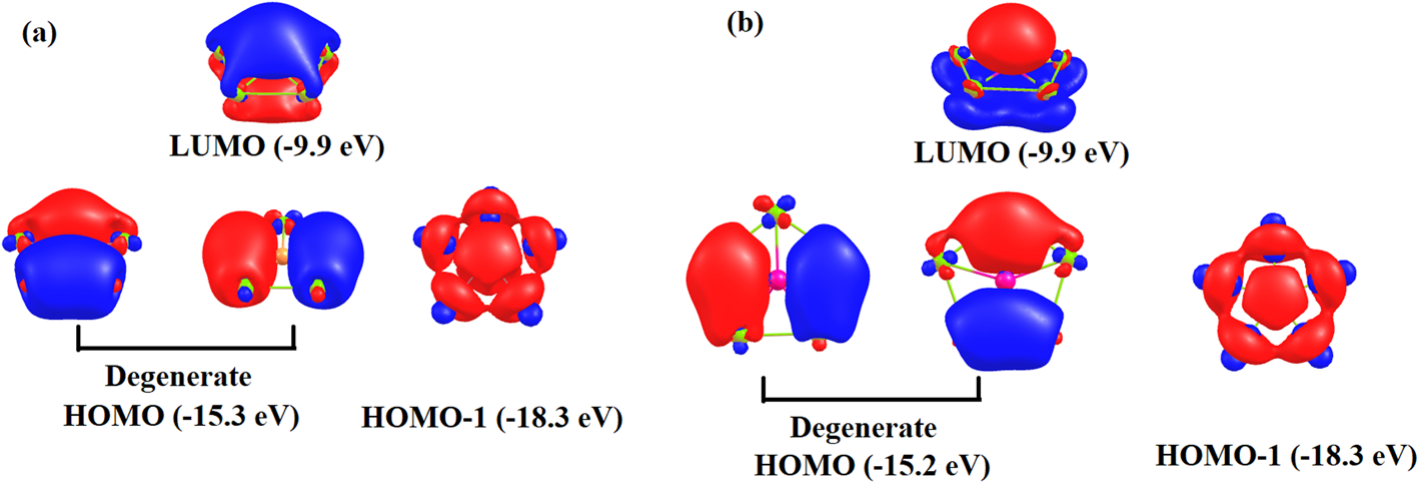
Frontier Kohn–Sham orbitals of (**a**) Cu_5_Al^2+^ and (**b**) Cu_5_Ga^2+^. Contour value used was 0.03 au.

The detailed electronic structure of the global minima has been studied using natural bonding orbital (NBO) analysis^44^. Figure 2 shows the frontier Kohn–Sham orbitals along with their energies in eV. The HOMO in each case is a doubly degenerate σ orbital consisting of Cu–Cu and Cu–Al/Ga bonds while the HOMO-1 encompasses the whole ring. These three sets of σ molecular orbitals (MOs) are responsible for showing σ-aromaticity according to Hückel (4n + 2) rule. However, there is no occupied π MOs which suggests absence of any π-aromaticity in these clusters. The calculated HOMO–LUMO gaps of these clusters are significant which may account for the stability of the singlet spin states of these clusters. The electronic structure of these clusters is exactly similar to that of σ-aromatic Au_5_Zn^+^ cluster^53^.

**Figure 3:**
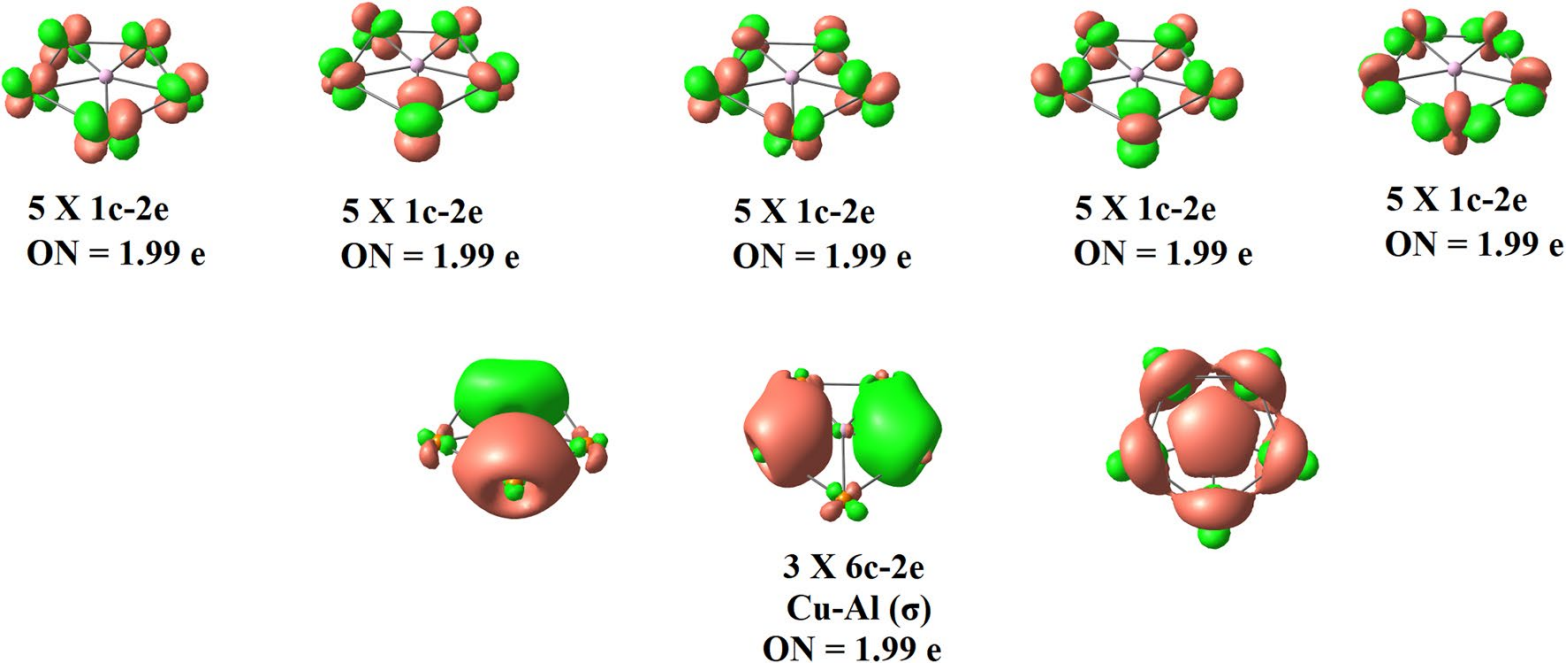
Bonds recovered using AdNDP analysis of Cu_5_Al^2+^ cluster.

**Figure 4:**
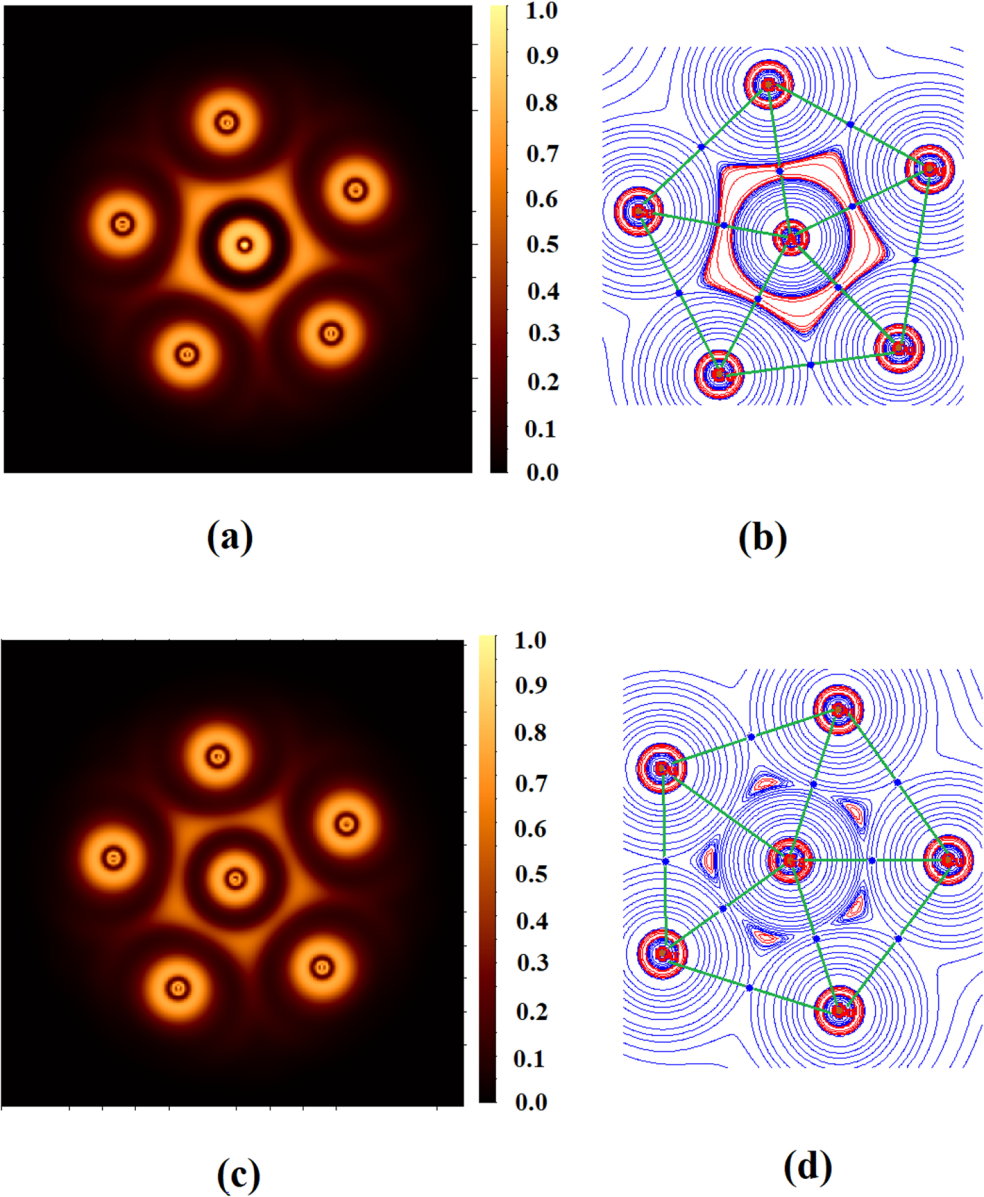
Plot of (**a**) laplacian of electron density (red = charge concentration, blue = charge depletion) and (**b**) electron localization function (ELF) in the molecular plane of Cu_5_Al^2+^ and (**c**) and (**d**) are for Cu_5_Ga^2+^.

We then turned our attention to investigate the electronic structure of the ppAl and ppGa structures using adaptive natural density partitioning (AdNDP) scheme^47^. AdNDP partitions the electron density in n-centre n-electron bonds and is very helpful in understanding the presence of multicentre bonds^47^. Figure 3 shows the bonds recovered using AdNDP analysis of Cu_5_Al^2+^ cluster as a representative case. AdNDP locates five d orbitals (1c–2e) on each Cu atom with occupation number of 1.99 |e| and three 6c–2e Cu–Al σ bonds which may result in σ-aromaticity in the ring. AdNDP analysis reveals the absence of π bonds and hence, certifies the absence of π aromaticity. The nature of Cu–Al and Cu–Ga bonds have been further analyzed within the realm of quantum theory of atoms in molecules (QTAIM)^48^ and electron localization function (ELF)^49,50^. The Cu-Al and Cu-Ga bonds are characterized by significant presence of electron density *ρ* (0.04 a.u) at the bond critical points, negative value of Laplacian of electron density ∇^2^*ρ*, negative value of local electronic energy density, *H*(r) and significant value of electron localization function (ELF) (Table 1). All these topological parameters refer to covalent character of the Cu–Al and Cu–Ga bonds. The Laplacian plot of electron density and electron localization function (Fig. 4) in the molecular plane clearly reveals significant electron delocalization.

**Table 1:** Electron density (*ρ*) at the bond critical points, Laplacian of electron density (*∇*ρ^2^), total electronic energy density (*H*(r)) and natural charges (q) in |e|.

Molecule	bcp	*ρ*	∇*ρ*^2^	*H*(r)	ELF	qAl	qGa	qCu
Cu_5_Al^2+^	Cu–Al bcp	0.048	− 0.022	− 0.012	0.67	− 1.28		0.65
Cu_5_Ga^2+^	Cu–Ga bcp	0.038	− 0.027	− 0.012	0.38		− 1.37	0.46

All other values are in a.u.

**Figure 5:**
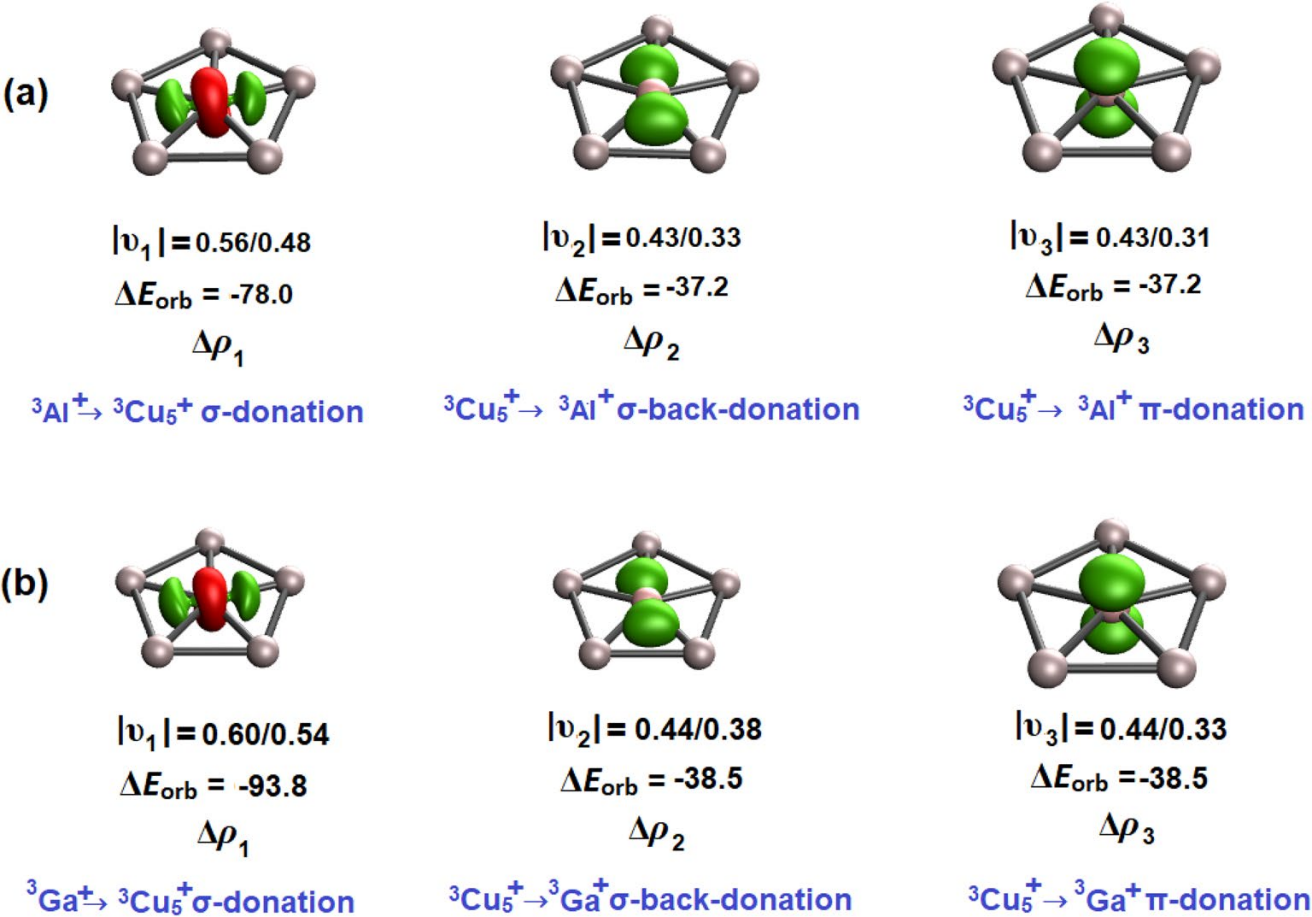
Plot of deformation density, Δρ obtained from ETS-NOCV analysis using Al^+^ and Ga^+^ (triplet) and Cu_5_^+^ in triplet state for (**a**) Cu_5_Al^2+^ and (**b**) Cu_5_Ga^2+^ clusters. Orbital values are given in kcal/mol. |υ| represents the (alpha/beta) charge eigenvalues. The direction of charge flow is red → green. Cut-off employed, Δρ = 0.004.

Again, to further quantify the strength of Cu–Al and Cu–B bonds, we carried out extended-transition state method for energy decomposition analysis combined with natural orbital of chemical valence (ETS-NOCV) (Fig. 5)^45,46^. The ETS-NOCV analysis suggests significant covalent nature of Cu–Al and Cu–Ga bonds. The NOCV pair densities were generated by considering two fragments, Al^+^ and Ga^+^ in triplet state and Cu_5_^+^ in triplet state. Suppl. Table S2 provides the numerical results of ETS-NOCV considering Al, Ga and Cu_5_ in different charges and electronic states as interacting fragments. Inspection of the relative size of Δ*E*_orb_ value reveal that the most reasonable fragmentation scheme is Al and Ga in cationic triplet state with ns^1^np^1^_⊥_ forming an electron-sharing π bond with the triplet Cu_5_^+^ state. This fragmentation provides the best description of ETS-NOCV as these fragments give the lowest Δ*E*_orb_ value. Apart from ^3^Al^+^/^3^ Ga^+^ → ^3^Cu_5_^+^ σ-donation, a significant amount of ^3^Cu_5_^+^ → ^3^Al^+^/^3^Ga^+^ σ-backdonation is also evident in the analysis. In addition, a significant amount of ^3^Cu_5_^+^ → ^3^Al^+^/^3^Ga^+^ π-donation is also found. These ^3^Cu_5_^+^ → ^3^Al^+^/^3^Ga^+^ donations account for the negative charges at central Al and Ga atoms.

**Figure 6:**
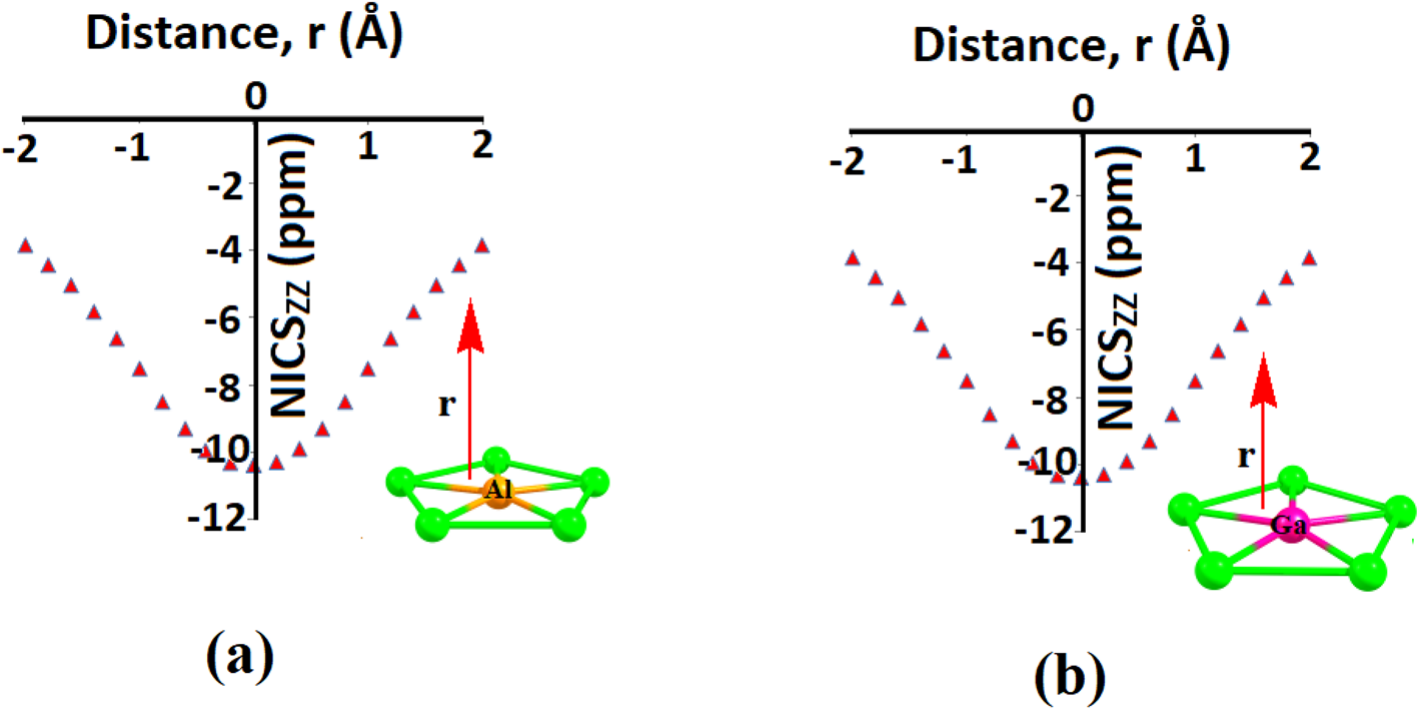
NICS_ZZ_ (ppm) against the perpendicular distance (Å) from the centre of the (**a**) Cu–Al–Cu in Cu_5_Al^2+^ cluster and (**b**) Cu–Ga–Cu in Cu_5_Ga^2+^ cluster.

For the quantify the aromaticity in the ppAl and ppGa global minima, we have performed nucleus independent chemical shift (NICS)^52^ calculations. NICS calculations are shown in Fig. 6. Significant negative values of the NICS_ZZ_ are found in the molecular plane and upto 2 Å above the molecular plane. This suggests the presence significant diatropic ring current and hence aromaticity in these clusters. The presence of σ-aromaticity has also been reported for planar tetracoordinate fluorine atom where “localization” played a vital role than delocalization^54^. Similar situation has also been reported of ppSi and ppGe clusters^55^.

**Figure 7:**
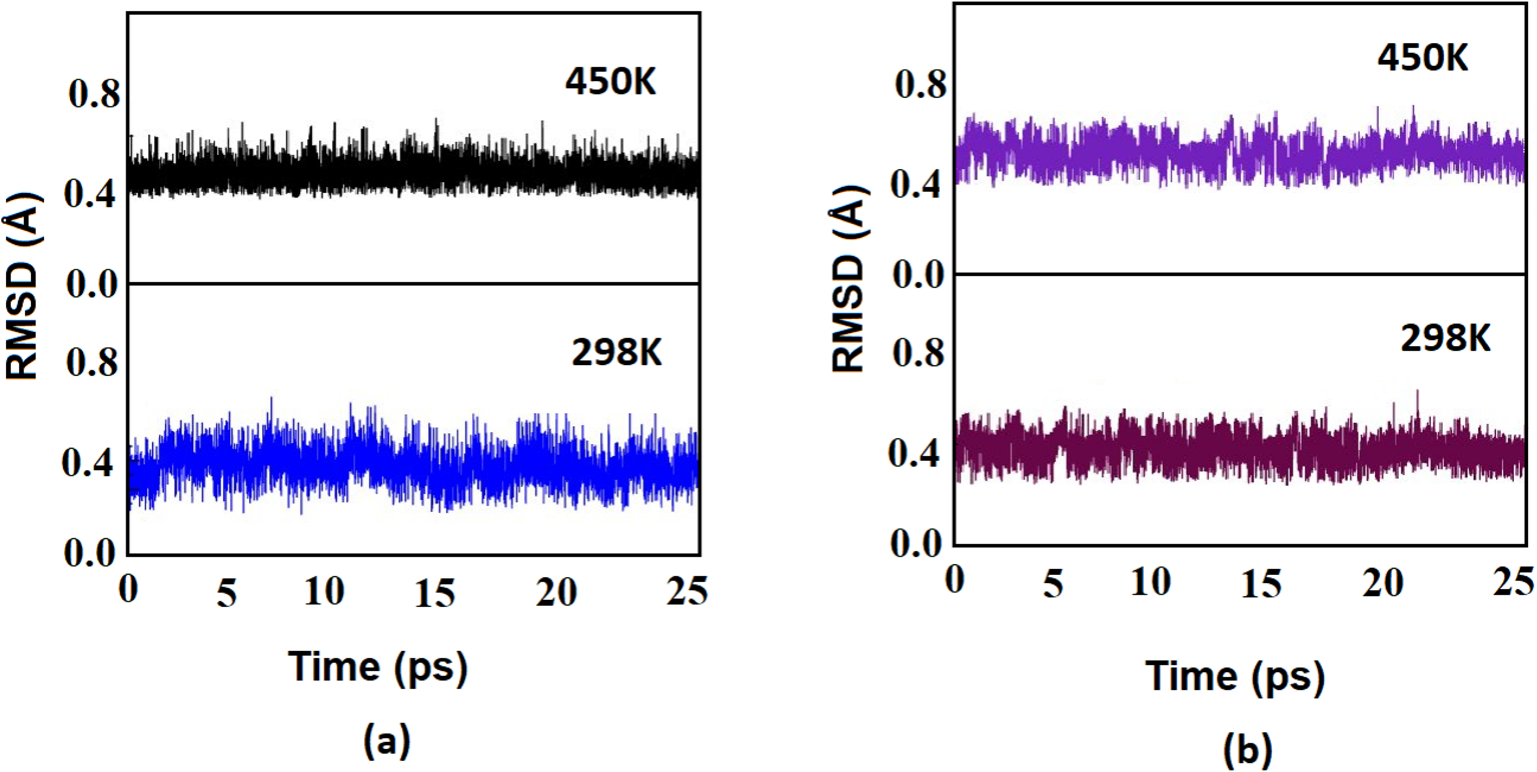
Plot of RMSD (Å) versus time (ps) obtained from BOMD simulation (M06-2X/TZVP) at 298 K and 450 K for (**a**) Cu_5_Al^2+^ and (**b**) Cu_5_Ga^2+^ clusters.

To investigate the dynamic stability of these clusters, we performed Born–Oppenheimer molecular dynamics (BOMD) simulations for a time period of 25 ps (Fig. 7). with a step size of 0.5 fs from the equilibrium global minimum structure with random velocities assigned to the atoms according to a Maxwell–Boltzmann distribution for one temperature, and then normalized so that the net moment for the entire system is zero. The BOMD calculations were performed at room temperature (298 K) and at elevated temperature (450 K). Figure 7 reveals that these ppAl and ppGa clusters are stable even at elevated temperature within a time frame of 25 ps.

## Conclusion

In summary, quantum chemical calculations predict the planar petacoordinate Al and Ga centres in Cu_5_Al^2+^ and Cu_5_Ga^2+^ are the global minima. These clusters are found to possess σ-aromaticity which may render stability to the planar form. These planar clusters are thermodynamically and kinetically very stable. Planar pentacoordination of other heavier group 13 elements such as In and Tl are not found. Although, the planar form of Cu_5_In^2+^ is true local minimum, however, it lies 3.4 kcal/mol higher than the global one (Suppl Figure S1). Planar form of Cu_5_Tl^2+^ is a higher order saddle point on the potential energy surface. Thus, it seems that Cu_5_ framework provides the best cavity size and electronic feature to stabilize planar form of Al and Ga as they have similar size. We feel that these clusters may be a suitable target for experimental characterization.

## Supplementary Information

Supplementary Information.

## Data Availability

All the DATA for this work is available with the corresponding authors on reasonable request.
