# Supplementary material for: σ-Aromaticity in planar pentacoordinate aluminium and gallium clusters
Source: Sci Rep. 2022 Jun 16;12:10041. doi: 10.1038/s41598-022-14430-4 (PMC9203769; doi:10.1038/s41598-022-14430-4)
Supplement: Supplementary file 1 — Supplementary Information. [file 41598_2022_14430_MOESM1_ESM.doc]

**Supporting Information**

**Of**

σ-Aromaticity in Planar Pentacoordinate Aluminium and Gallium Clusters

Amlan J. Kalita,^a^ Kangkan Sarmah,^a^ Farnaz Yashmin,^a^ Ritam R. Borah,^a^ Indrani Baruah,^a^ Rinu P. Deka^a^ and Ankur K. Guha*^a^

Advanced Computational Chemistry Centre, Department of Chemistry, Cotton University, Panbazar, Guwahati, Assam, INDIA-781001

^*^Email: [ankurkantiguha@gmail.com](mailto:ankurkantiguha@gmail.com)

**
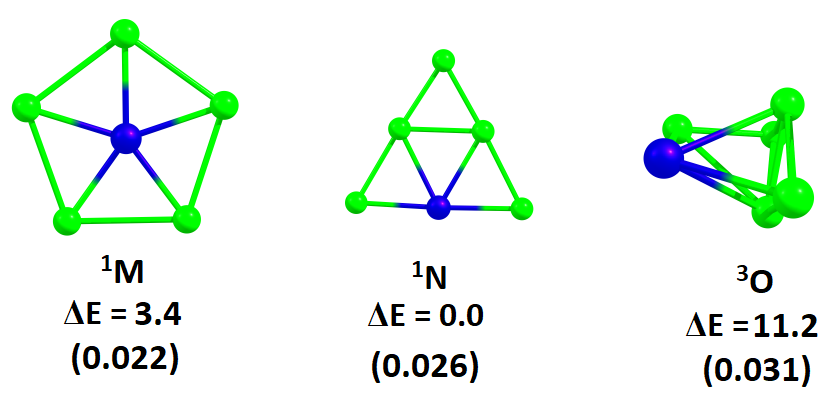
**

**Figure S1.** The relative energies in kcal/mol of the low energy isomers of Cu_5_In^2+^ calculated at CCSD(T)/def2-TZVP//M06-2X/def2-TZVP level of theory. T_1_ diagnostic values are given within parenthesis.

**Table S1**. Calculated results for ppAl and ppGa local minima at different level along with their lowest vibrational frequency, ν_min_ (cm^-1^).

| **Structure** | **Level** | **ν_min_** |
| --- | --- | --- |
| ppAl | TPSSh/def2-TZVP | 50.0 |
|  | PBE0/def2-TZVP | 48.5 |
| ppGa | TPSSh/def2-TZVP | 49.8 |
|  | PBE0/def2-TZVP | 47.2 |

**Table S2. ETS-NOCV results (Δ*E*_orb_ in kcal/mol) of Cu_5_Al^2+^ and Cu_5_Ga^2+^ clusters considering Al, Ga and Cu_5_ in different charge and electronic states as interacting fragments at M06-2X/def2-TZVP level.**

|  |  | Cu_5_Al^2+^ | | |
| --- | --- | --- | --- | --- |
| Cluster |  | Al^+^ (T, 3s^1^3p_z_^1^)  + Cu_5_^+^ (T) | Al (D, 3s^2^3p_z_^1^)  + Cu_5_^2+^ (D) | Al^2+^ (D, 3s^1^)  + Cu_5_ (D) |
|  | Δ*E*_orb_ | -152.4 | -182.3 | -176.2 |
|  |  | Cu_5_Ga^2+^ | | |
|  |  | Ga^+^ (T, 4s^1^4p_z_^1^)  + Cu_5_^+^ (T) | Ga (D, 4s^2^4p_z_^1^)  + Cu_5_^2+^ (D) | Ga^2+^ (D, 4s^1^)  + Cu_5_ (D) |
|  | Δ*E*_orb_ | -170.8 | -202.4 | -192.3 |

**Cartesian coordinates of all the molecules calculated at M06-2X/def2-TZVP level and their energies (in au) at CCSD(T)/def2-TZVP without ZPVE.**

**^1^A**

**E_CCSD(T)_ = -8438.12304**

13 0.000000000 0.000442000 0.000000000

29 2.225575000 1.098270000 0.000000000

29 -1.154511000 -2.196635000 0.000000000

29 -2.445955000 0.419055000 0.000000000

29 -0.357362000 2.455999000 0.000000000

29 1.732252000 -1.776888000 0.000000000

**^1^B**

**E_CCSD(T)_ = -8438.11045**

29 -0.701164000 -1.360394000 0.006444000

29 -0.701327000 1.360322000 0.006343000

29 1.834291000 -2.490661000 -0.002462000

29 1.833936000 2.490907000 -0.002407000

13 1.483064000 0.000047000 -0.002683000

29 -2.930558000 -0.000195000 -0.006715000

**^1^C**

**E_CCSD(T)_ = -8438.09051**

29 2.379466000 -1.065760000 0.000000000

29 0.000000000 0.176467000 0.000000000

29 -2.379472000 -1.065742000 0.000000000

29 2.243656000 1.524353000 0.000000000

29 -2.243646000 1.524372000 0.000000000

13 -0.000009000 -2.439772000 0.000000000

**^1^D**

**E_CCSD(T)_ = -8438.08934**

29 0.264634000 -0.519989000 -1.242708000

29 -2.080675000 -1.111076000 0.000357000

29 2.662037000 -0.221688000 0.000244000

29 -1.556484000 1.491888000 -0.000499000

29 0.264320000 -0.518342000 1.242971000

13 0.995297000 1.961308000 -0.000814000

**^3^E**

**E_CCSD(T)_ = -8438.07758**

13 0.000018000 -0.239622000 -1.270106000

29 -2.249280000 -1.123967000 -0.290424000

29 2.249278000 -1.124002000 -0.290371000

29 -0.000016000 -0.529140000 1.241784000

29 -1.516330000 1.442245000 -0.045787000

29 1.516340000 1.442281000 -0.045845000

**^3^F**

**E_CCSD(T)_ = -8437.98810**

29 -2.372023000 1.478032000 -0.004817000

29 1.039763000 1.059947000 0.005175000

29 -3.455282000 -1.042639000 0.002578000

29 1.549571000 -1.432752000 -0.003490000

13 -0.955416000 -0.603746000 0.002182000

29 3.666260000 0.208057000 -0.000424000

**^1^G**

**E_CCSD(T)_ = -10119.60023**

31 0.002053000 0.001558000 0.010886000

29 -2.018051000 -1.474011000 -0.003815000

29 2.502188000 0.006677000 -0.000514000

29 -2.030600000 1.458309000 0.000289000

29 0.764614000 2.382756000 -0.005059000

29 0.779654000 -2.375396000 -0.002538000

**^1^H**

**E_CCSD(T)_ = -10119.58664**

29 0.871018000 1.352596000 0.000225000

29 0.870810000 -1.352717000 0.000025000

29 -1.676814000 2.515706000 -0.000074000

29 -1.677263000 -2.515467000 0.000034000

31 -1.394150000 0.000113000 -0.000076000

29 3.102548000 -0.000237000 -0.000129000

**^1^I**

**E_CCSD(T)_ = -10119.57109**

29 -0.803147000 2.400456000 0.000000000

29 0.434698000 0.000000000 0.000000000

29 -0.803147000 -2.400456000 0.000000000

29 1.778208000 2.245072000 0.000000000

29 1.778208000 -2.245071000 0.000000000

31 -2.230959000 0.000000000 0.000000000

**^1^J**

**E_CCSD(T)_ = -10119.56930**

29 0.052994000 -0.773356000 -1.244191000

29 -2.359901000 -0.956837000 0.000131000

29 2.483274000 -0.786978000 0.000998000

29 -1.467644000 1.535715000 -0.000570000

29 0.051688000 -0.769903000 1.244853000

31 1.159615000 1.638369000 -0.001142000

**^3^K**

**E_CCSD(T)_ = -10119.55006**

31 -0.248138000 -0.008214000 0.660649000

29 1.287160000 1.349505000 -0.989114000

29 1.299114000 -1.345206000 -0.995921000

29 -2.255426000 1.380929000 0.008805000

29 -2.269040000 -1.376388000 0.008129000

29 2.203444000 -0.000059000 1.261890000

**^3^L**

**E_CCSD(T)_ = -10119.54406**

29 -1.485504000 -1.275404000 -0.000560000

29 -1.484809000 1.275131000 -0.000482000

29 2.990155000 -1.353891000 0.000220000

29 2.988436000 1.354844000 0.000286000

31 0.814179000 -0.000886000 -0.000212000

29 -3.878608000 0.000268000 0.000763000
